# Right ventricular infarction with a culprit lesion in the distal right coronary artery: incidence and clinical characteristics

**DOI:** 10.20407/fmj.2025-036

**Published:** 2026-09-25

**Authors:** Yuki Takigawa, Naoki Hoshino, Meiko Hoshino, Akira Yamada, Masanobu Yanase, Hideo Izawa

**Affiliations:** Department of Cardiology, Fujita Health University, School of Medicine, Toyoake, Aichi, Japan

**Keywords:** Coronary vessels, Global longitudinal strain, ST elevation myocardial infarction, Percutaneous coronary intervention, Echocardiography

## Abstract

**Objectives:**

Right ventricular infarction (RVI) is commonly observed in patients with inferior ST-elevation myocardial infarction and typically results from proximal right coronary artery (RCA) occlusion. However, the occurrence of RVI and its clinical characteristics in patients with distal RCA lesions are not well defined.

**Methods:**

We retrospectively investigated 128 patients with inferior ST-elevation myocardial infarction caused by RCA occlusion who underwent primary percutaneous coronary intervention at our institution between January 2020 and December 2023. RVI was diagnosed based on echocardiographic evidence and hemodynamic instability. Clinical characteristics and incidence rates were compared between patients with RVI [the RVI (+) group] and those without RVI [the RVI (–) group]. Findings on coronary angiography were assessed in detail for patients with distal RCA lesions.

**Results:**

RVI was identified in 24/128 patients (18.8%), including 3/31 patients (9.7%) with distal RCA lesions. Right ventricular global longitudinal strain (RVGLS) was significantly lower in the RVI (+) group (–14.0% [–16.4, –11.6] vs. –16.4% [–18.7, –13.9]; *p*=0.004), as was right ventricular free wall strain (RVFWS) (–14.7% [–18.5, –10.6] vs. –17.7% [–20.7, –15.0]; *p*=0.01). RVGLS and RVFWS values were comparable in the RVI (+) group regardless of whether the RCA lesion was distal or proximal. There was no significant difference in TIMI flow before or after percutaneous coronary intervention or in the distribution of lesions.

**Conclusions:**

RVI can be caused by distal RCA lesions, albeit less often than by proximal RCA lesions. No significant differences in RV strain parameters were observed between distal and proximal lesions.

## Introduction

Right ventricular infarction (RVI) occurs in approximately half of all patients with acute inferior ST-elevation myocardial infarction (STEMI) and is associated with increased in-hospital mortality.^1^ RVI impairs right ventricular (RV) function, leading to hemodynamic instability by reducing left ventricular preload and contributing to systemic hypotension.^2^ RVI requires early diagnosis and treatment to optimize patient outcomes.^1,3^ RVI is typically attributed to occlusion of the proximal right coronary artery (RCA), which supplies the RV free wall through its RV branches.^3,4^ However, cases of RVI caused by distal RCA occlusion are occasionally encountered in clinical practice. Although there has been some research on the incidence and clinical characteristics of RVI in distal RCA lesions,^4^ the evidence remains limited.

Advances in echocardiographic techniques, particularly strain imaging, have improved the assessment of RV function.^5–7^ Previous studies have identified RV global longitudinal strain (RVGLS) and RV free wall strain (RVFWS) to be reliable indicators of RV dysfunction in patients with STEMI.^8,9^ Therefore, we hypothesized that RV strain parameters, specifically RVGLS and RVFWS, could quantitatively characterize RV dysfunction in patients with distal RCA lesions who develop RVI.

The aim of this study was to determine the incidence and clinical characteristics of RVI in patients with STEMI and a culprit lesion in the distal RCA.

## Methods

### Study population

This study had a single-center, retrospective, observational design and was performed at Fujita Health University Hospital. All patients with STEMI who underwent emergency percutaneous coronary intervention (PCI) for a culprit lesion in the RCA between January 2020 and December 2023 were identified by a search of the electronic medical records.

The following exclusion criteria were applied: previous coronary artery disease, including pre-PCI and post-PCI coronary artery bypass grafting, myocardial disease, history of tricuspid valve or right-sided heart surgery, including tricuspid annuloplasty, transthoracic echocardiography (TTE) not performed within 24 h of emergency PCI for inferior STEMI or TTE images of insufficient quality for evaluation, presence of cardiac tamponade, and active pacing during TTE.

Data were extracted from the medical records, including patient demographics, the clinical course, laboratory results, findings on coronary angiography (CAG) and PCI, and echocardiographic assessments performed within 24 h of emergency PCI for inferior STEMI. CAG images for patients with RVI and a culprit lesion in the distal RCA were analyzed in detail.

### Definition of right ventricular infarction

For the purposes of this study, RVI was defined as RV wall hypokinesis on echocardiography, accompanied by hemodynamic instability attributable to RVI, such as the need for intravenous volume resuscitation beyond standard protocols or use of catecholamines, in the absence of alternative causes such as sepsis or active bleeding. The right-sided precordial leads (V3R/V4R) were not routinely recorded in the majority of patients during the study period; therefore, electrocardiographic findings from the right-sided leads were not included into the diagnostic criteria. Considering that variable definitions of RVI have been used in previous studies,^1,3,4^ we used a definition that emphasizes clinically significant RVI requiring therapeutic intervention.

### Coronary angiography and PCI

All patients underwent PCI according to the most recent American College of Cardiology/American Heart Association guidelines at the time of treatment.^10^ The culprit lesion was first dilated using a balloon, followed by stent deployment in accordance with the operator’s clinical judgment. All patients received antiplatelet therapy, including aspirin and glycoprotein IIb/IIIa receptor antagonists. CAG was performed before and after PCI to assess the severity of stenosis and TIMI (Thrombolysis in Myocardial Infarction) flow grade.

The culprit lesion in the RCA was classified according to whether it was in segment 1, 2, 3, or 4 or in the posterolateral branch. In this study, the distal RCA was defined as segments 3 and 4 and the posterolateral branch.

We analyzed the findings on CAG in more detail for patients with a culprit lesion in the distal RCA, specifically evaluating RV branch slow flow, coronary dominance, and collateral circulation in the right ventricle. RV branch slow flow was defined as a TIMI flow grade of 0, 1, or 2 in at least one RV branch.

### Echocardiographic assessment

TTE was performed within 24 h of emergency PCI for inferior STEMI using a Vivid E95 ultrasound system (GE Healthcare, Chicago, IL, USA). An experienced sonographer acquired the images, all parameters of which were subsequently analyzed by a certified echocardiologist. RV wall motion abnormalities were independently reassessed by a second experienced echocardiologist who was blinded to the clinical data. Disagreements were resolved by consensus.

RV function was assessed by visual evaluation of gross wall motion abnormalities (apical four-chamber or RV-focused apical view). Quantitative measures including RVGLS, RVFWS, and tricuspid annular plane systolic excursion (TAPSE) were also recorded. TAPSE was measured using M-mode echocardiography.

RVGLS and RVFWS were assessed using speckle-tracking echocardiography with dedicated software in accordance with the American Society of Echocardiography guidelines.^11^ RVGLS was derived from the mean strain of three RV free wall segments and three septal segments. RVFWS was calculated from the mean strain of the three RV free wall segments alone. To ensure consistency, all analyses were performed by the echocardiologist who obtained the measurements.

We also compared echocardiographic parameters between patients with RVI from proximal RCA lesions and those with RVI from distal RCA lesions to assess potential differences in RV dysfunction.

### Statistical analysis

Continuous variables are expressed as the mean±standard deviation if normally distributed and as the median (interquartile range [IQR]) if not normally distributed. Categorical variables are shown as the count (percentage). Continuous data were compared between groups using the Student’s *t*-test or Mann–Whitney *U* test and categorical data using the chi-squared test or Fisher’s exact test depending on the data distribution. All statistical analyses were performed using JMP version 13.0 (SAS Institute Inc., Cary, NC, USA). A *p*-value of <0.05 was considered statistically significant.

## Results

### Patient characteristics

A total of 170 patients underwent emergency PCI for inferior STEMI with a culprit lesion in the RCA during the study period. After exclusion of 31 patients with prior coronary artery disease, 6 who lacked timely TTE (within 24 h post-PCI), and 5 with inadequate image quality, the final cohort included 128 patients. Notably, no patient was excluded based on prespecified criteria, such as previous tricuspid valve surgery, severe tricuspid regurgitation, or pericardial effusion exceeding physiological levels. Twenty-four patients (18.8%) in the cohort had RVI [the RVI (+) group] and 104 (81.2%) did not [the RVI (–) group].

The baseline characteristics of the study population are summarized in Table 1. The mean age was 70±12 years, and 66.4% were male. There were no statistically significant between-group differences in vital signs measured at the initial clinical contact (mostly on arrival in the emergency room), including systolic and diastolic blood pressure and heart rate, or in laboratory results (hemoglobin, creatinine, C-reactive protein, N-terminal pro B-type natriuretic peptide, cholesterol, troponin). Complete atrioventricular block and temporary use of a pacemaker were slightly more common in the RVI (+) group; however, these differences were not statistically significant. Notably, the median creatine kinase–myocardial band value at baseline was significantly lower in the RVI (+) group than in the RVI (–) group (2.3 ng/mL [IQR 1.0, 2.7] vs. 3.6 ng/mL [IQR 2.0, 5.0]; *p*<0.001).

### Findings on CAG and PCI

As shown in Table 2, the distribution of culprit lesions was comparable between the RVI (+) and RVI (–) groups. Lesions in segment 3 and the posterolateral branch were less common in the RVI (+) group; these differences were not statistically significant. Similarly, there was no statistically significant between-group difference in pre-PCI TIMI flow grade, although a trend toward lower TIMI grades was observed in the RVI (+) group. The distribution of post-PCI TIMI grades was also comparable between the two groups. All 31 patients with distal RCA lesions had RCA-dominant coronary anatomy with a complete absence of RV collateral circulation. The CAG findings for patients with a culprit lesion in the distal RCA are summarized in Table 3. RVI was observed in three of these 31 cases (9.7%). While pre-PCI RV branch slow flow was observed more often in the RVI (+) group, post-PCI RV branch slow flow was more common in the RVI (–) group. These differences were not statistically significant. Representative coronary angiograms for a patient with RVI attributable to a distal RCA lesion and for a patient without RVI are shown in Figure 1, illustrating the contrast in RV branch flow before and after PCI.

### Echocardiographic findings

The echocardiographic findings are compared between the RVI (+) and RVI (–) groups in Table 4. RVGLS and RVFWS values were significantly lower (less negative) in the RVI (+) group than in the RVI (–) group. Furthermore, the RVI (+) group had a significantly higher TAPSE value and a significantly lower left ventricular ejection fraction. The inferior vena cava diameter was also larger in the RVI (+) group. However, there was no significant between-group difference in the tricuspid regurgitation pressure gradient.

As shown in Table 5, RVGLS and RVFWS values were similarly reduced (less negative) whether RVI was in the proximal or distal RCA. There were no statistically significant between-group differences in the echocardiographic parameters.

## Discussion

This study explored the incidence and clinical characteristics of RVI in patients who had inferior STEMI and a culprit lesion in the distal RCA. RVI was present in approximately 24.0% of all cases with inferior STEMI and in 9.7% of those with distal RCA lesions. These findings are in line with previous studies reporting RVI in 30%–50% of cases with inferior STEMI, although differences in diagnostic criteria may account for variations in reported incidence rates.^1–3^

Critically, our results confirm that RVI can occur in patients with distal RCA occlusion and align with those of Bowers et al.,^4^ who focused on the physiological fact that the RV free wall is supplied by RV branches arising from the RCA, including the acute marginal branches, and demonstrated that occlusion compromising flow in these branches can lead to RVI even in the setting of distal RCA lesions. However, previous studies have primarily emphasized proximal RCA occlusions.^2,3,5^ In our study, all patients with RVI caused by distal RCA lesions were found to have pre-PCI RV branch slow flow, and none had slow flow post-PCI. Conversely, approximately 50% of patients without RVI showed post-PCI RV branch slow flow, likely because of stent jailing. These findings indicate that while pre-PCI RV branch slow flow may reflect ischemic injury contributing to RVI, slow flow after PCI appears to be clinically insignificant in this setting.

Echocardiographic findings further confirmed RV dysfunction in patients with RVI. RVGLS and RVFWS were significantly lower in our RVI (+) group, consistent with previous studies demonstrating impaired RV myocardial deformation in this population.^6–9^ In the RVI (+) group, there was no significant difference in RVGLS or RVFWS according to whether the culprit lesion in the RCA was proximal or distal, suggesting that if RVI develops, the degree of RV dysfunction is comparable regardless of the location of the lesion. Interestingly, TAPSE was paradoxically higher in the RVI (+) group, which may be attributed to the small sample size and warrants further investigation. These findings align with recent research emphasizing the diagnostic and prognostic value of speckle-tracking-derived strain parameters in the assessment of RV function in acute myocardial infarction.^9,12^

Although we analyzed CAG images in detail to identify anatomical predictors of RVI, such as TIMI flow grade, RV branch involvement, and coronary dominance, we could not identify a reliable angiographic marker that could predict RVI in patients with a distal RCA lesion. While previous studies have suggested potential anatomical correlations,^4^ our findings underscore the limited predictive value of CAG alone.

This study has some limitations. The retrospective single-center design and the relatively small sample of distal RCA cases with RVI limit the generalizability of our findings. Furthermore, right-sided precordial leads were not routinely recorded in the majority of patients during the study period. Therefore, RVI defined by electrocardiographic criteria may have been underdiagnosed. Nevertheless, our study contributes to the growing body of literature supporting the use of strain-based echocardiographic assessments in RVI. Its findings also underscore the importance of recognizing RVI, even in cases with distal RCA lesions.

In conclusion, RVI can occur in patients with a distal RCA lesion, albeit less often than in those with a proximal lesion. In this small cohort, no statistically significant difference in RV strain parameters was observed between proximal and distal RCA culprit lesions after development of RVI. However, given the very small number of distal cases (n=3), the study was underpowered to detect potential differences. Larger studies are needed to clarify whether RV functional indices differ according to the location of the culprit lesion.

## Figures and Tables

**Figure 1:**
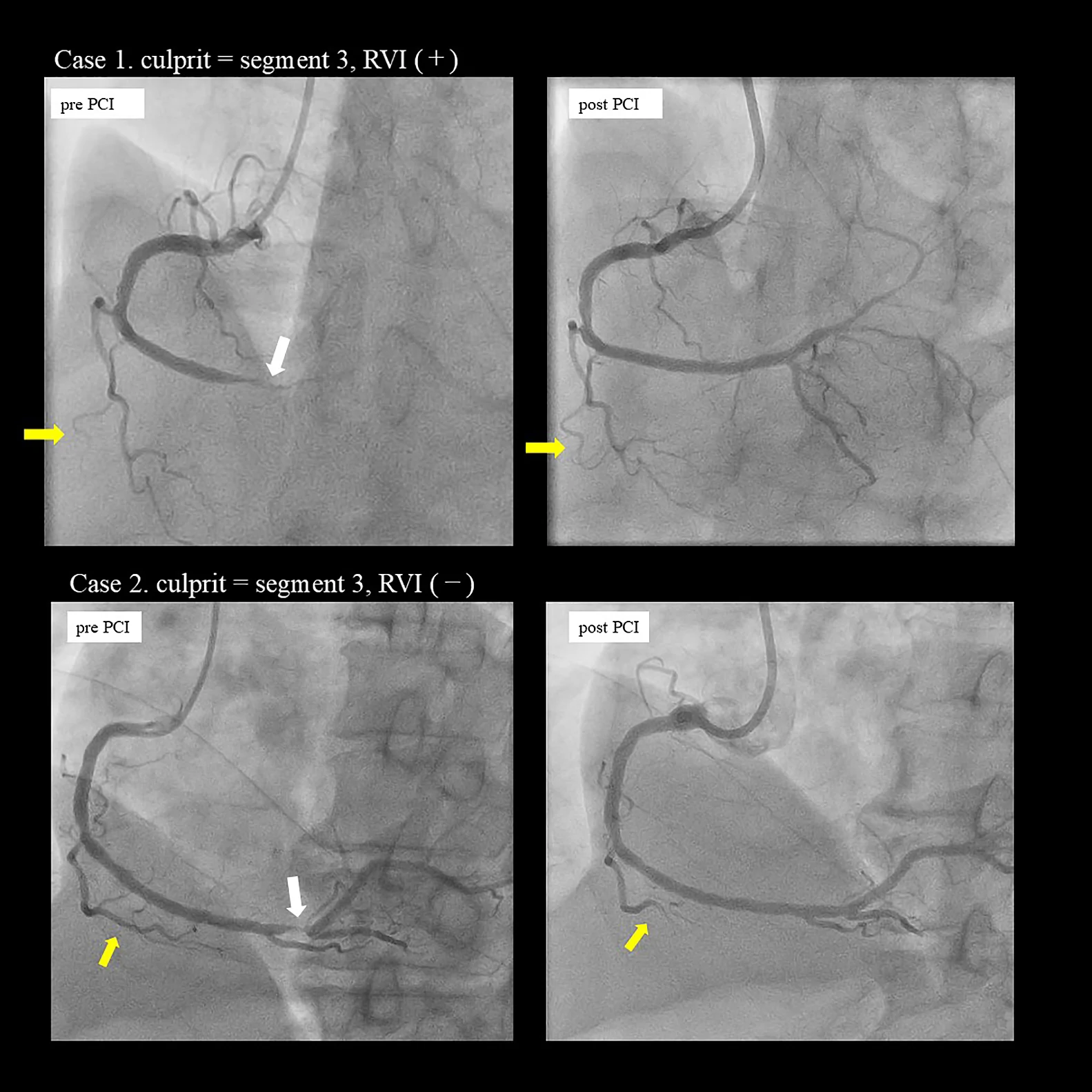
Representative coronary angiograms of patients with distal RCA culprit lesions with and without RVI. Case 1. Findings on coronary angiography for a patient with RVI and a culprit lesion in the distal RCA. Pre-PCI (left) and post-PCI (right) right coronary angiograms are shown. The culprit lesion is located in segment 3 of the RCA (white arrows), with slow flow in the RV branch (yellow arrows) observed pre-PCI. After successful PCI, coronary flow is restored, and RV branch perfusion improves. Case 2. Findings on coronary angiography for a patient without RVI and a distal RCA culprit lesion. Pre-PCI (left) and post-PCI (right) right coronary angiograms are shown. The culprit lesion was located in segment 3 of the RCA (white arrows). No slow flow was observed in the RV branch (yellow arrows) pre-PCI. However, post-PCI angiography revealed emergence of slow flow in the RV branch. PCI, percutaneous coronary intervention; RCA, right coronary artery; RV, right ventricular; RVI, right ventricular infarction.

**Table 1:** Demographic, clinical, medication, and laboratory data

	N	Overall (n=128)	RVI (+) group (n=24)	RVI (–) group (n=104)	*p*-value
Demographic and clinical data					
Age, years	128	70±12	72±11	70±13	0.440
Male	128	85 (66.4)	13 (54.1)	72 (69.2)	0.159
Body mass index*	128	23.1 [20.0, 26.0]	24.4 [22.0, 26.7]	22.7 [20.0, 26.0]	0.115
Systolic BP, mmHg	128	133±33	130±31	134±33	0.521
Diastolic BP, mmHg	128	78±23	77±22	78±23	0.793
Heart rate, bpm	128	71±22	74±23	70±22	0.426
Complete AV block	128	18 (14.0)	3 (12.5)	21 (19.0)	0.080
Temporary pacemaker	128	41 (32.0)	10 (41.7)	31 (29.8)	0.261
VT/VF	128	12 (9.3)	3 (12.5)	9 (8.7)	0.560
IABP support	128	25 (19.5)	3 (12.5)	22 (21.1)	0.335
Impella^TM^ support	128	1 (0.7)	0	1 (0.9)	0.629
VA-ECMO support	128	2 (1.5)	0	2 (1.9)	0.493
CV risk factors					
Hypertension	128	70 (54.7)	11 (45.8)	59 (56.7)	0.333
Dyslipidemia	128	48 (37.5)	7 (29.1)	41 (39.4)	0.349
Diabetes mellitus	128	36 (28.1)	6 (25.0)	30 (28.9)	0.705
Maintenance dialysis	128	1 (0.7)	1 (4.1)	0	0.659
Atrial fibrillation	128	3 (2.3)	0	3 (2.8)	0.399
Current smoking	127	32 (25.2)	8 (33.3)	24 (23.3)	0.308
Medication					
Aspirin	128	10 (7.8)	3 (12.5)	7 (6.7)	0.342
P2Y12 inhibitor	128	0	0	0	NA
Anticoagulant	128	2 (1.6)	0 (0.0)	2 (1.9)	0.493
ACE-I/ARB/ARNI	128	42 (32.8)	6 (25.0)	36 (34.6)	0.365
Beta-blocker	128	10 (7.8)	2 (8.3)	8 (7.7)	0.916
Statin	128	29 (22.7)	4 (16.7)	25 (24.0)	0.436
MRA	128	4 (3.1)	1 (4.1)	3 (2.9)	0.744
SGLT2 inhibitor	128	2 (1.6)	1 (4.1)	1 (0.9)	0.253
Laboratory data					
Hemoglobin, g/dL	128	11.8±1.7	11.4±1.9	11.8±1.7	0.223
Creatinine, mg/dL	128	0.89 [0.77, 1.12]	0.88 [0.77, 1.12]	0.90 [0.77, 1.12]	0.714
CRP, mg/dL	128	0.13 [0.06, 0.36]	0.14 [0.06, 0.28]	0.12 [0.06, 0.44]	0.807
NT-proBNP, pg/mL	128	281 [77, 820]	213 [109, 783]	308 [74, 820]	0.714
Log (NT-proBNP)	128	5.7±1.7	5.5±1.6	5.7±1.7	0.574
TC, mg/dL	128	163 [140, 181]	163 [137, 177]	163 [140, 184]	0.727
Triglycerides, mg/dL	128	37 [28, 49]	39 [32, 60]	36 [28, 48]	0.336
LDL-C, mg/dL	128	103 [87, 127]	96 [82, 124]	107 [87, 127]	0.302
HDL-C, mg/dL	128	38 [32, 43]	38 [31, 44]	38 [32, 43]	0.718
CK, baseline, U/L	128	77 [50, 117]	91 [60, 138]	74 [49, 111]	0.137
CK, peak, U/L	128	1679 [885, 2918]	1219 [614, 2327]	1822 [953, 3207]	0.172
CK–MB, baseline, ng/mL	128	2.9 [1.7, 4.6]	2.3 [1.0, 2.7]	3.6 [2.0, 5.0]	<0.001
CK–MB, peak, ng/mL	128	189.3 [97.2, 276.4]	149 [62, 249]	197 [99, 302]	0.295
Troponin I, baseline, ng/mL	128	0.02 [0.01, 0.08]	0.02 [0.01, 0.05]	0.02 [0.01, 0.08]	0.957
Troponin I, peak, ng/mL	128	64.3 [30.8, 171.3]	43.1 [20.2, 95.1]	72.2 [33.5, 184.0]	0.071

Data are shown as the mean±standard deviation, median [interquartile range], or count (percentage) as appropriate. *Calculated as kg/m^2^. ACE-I, angiotensin-converting enzyme inhibitor; ARNI, angiotensin receptor–neprilysin inhibitor; AV, atrioventricular; BP, blood pressure; CK, creatine kinase; CK–MB, creatine kinase–myocardial band; CRP, C-reactive protein; CV, cardiovascular; HDL, high-density lipoprotein cholesterol; IABP, intra-aortic balloon pump; LDL, low-density lipoprotein cholesterol; NT-pro BNP, N-terminal pro B-type natriuretic peptide; RV, right ventricular; RVI, right ventricular infarction; SGLT2, sodium–glucose cotransporter 2; TC, total cholesterol; MRA, mineralocorticoid receptor antagonist; VA-ECMO, veno-arterial extracorporeal membrane oxygenation; VF, ventricular fibrillation; VT, ventricular tachycardia.

**Table 2:** Findings on coronary angiography and percutaneous coronary intervention

	N	Overall (n=128)	RVI (+) group (n=24)	RVI (–) group (n=104)	*p*-value
Site of culprit lesion	128				
Segment 1 (proximal RCA)		45 (35)	10 (42)	35 (34)	0.458
Segment 2 (mid RCA)		52 (41)	11 (46)	41 (39)	0.564
Segment 3 (distal RCA)		24 (19)	3 (12)	21 (20)	0.384
Segment 4 (PDA)		3 (2)	0	3 (3)	0.399
PL		4 (3)	0	4 (4)	0.329
Initial TIMI flow grade	128				
0		81 (63)	14 (58)	67 (65)	0.576
1		16 (13)	5 (21)	11 (10)	0.170
2		10 (8)	1 (4)	9 (9)	0.460
3		21 (16)	4 (17)	17 (16)	0.969
Final TIMI flow grade	128				
0		0	0	0	NA
1		0	0	0	NA
2		5 (4)	0	5 (5)	0.273
3		123 (96)	24 (100)	99 (95)	0.273
Multivessel coronary artery disease	128	57 (45)	10 (41)	47 (45)	0.753
Procedural success	128	126 (98)	24 (100)	102 (98)	0.360
Stent insertion	128	122 (96)	24 (100)	98 (94)	0.109

Data are shown as the number or number (percentage) as appropriate. PDA, posterior descending artery; PL, posterolateral branch; RCA, right coronary artery; RV, right ventricular; RVI, right ventricular infarction; TIMI, Thrombolysis in Myocardial Infarction.

**Table 3:** Comparison of coronary angiographic findings between patients with and without right ventricular infarction and distal RCA lesions

	N	All distal RCA (n=31)	RVI (+) group (n=3)	RVI (–) group (n=28)	*p*-value
Site of culprit lesion	31				
Segment 3 (distal RCA)		24 (78)	3 (100)	21 (75)	0.325
Segment 4 (PDA)		6 (19)	0	6 (21)	0.550
PL		1 (3)	0	1 (4)	0.483
Initial TIMI flow grade	31				
0		11 (36)	1 (33)	10 (36)	0.934
1		6 (19)	2 (67)	4 (14)	0.029
2		4 (13)	0	4 (14)	0.483
3		10 (32)	0	10 (36)	0.208
Final TIMI flow grade	31				
0		0	0	0	NA
1		0	0	0	NA
2		2 (6)	0	2 (7)	0.632
3		29 (94)	3 (100)	26 (93)	0.632
Slow flow in RV branch before PCI	31	25 (81)	3 (100)	22 (76)	0.371
Slow flow in RV branch after PCI	31	13 (42)	0	13 (46)	0.121

Data are shown as the number or number (percentage) as appropriate. PCI, percutaneous coronary intervention; PDA, posterior descending artery; PL, posterolateral branch; RCA, right coronary artery; RV, right ventricular; RVI, right ventricular infarction; TIMI, Thrombolysis in Myocardial Infarction.

**Table 4:** Comparison of echocardiographic parameters in all patients and according to whether or not RVI was present

	N	Overall (n=128)	RVI (+) group (n=24)	RVI (–) group (n=104)	*p*-value
Left ventricle					
LV end-diastolic volume, mL	126	86 [72, 108]	80 [71, 105]	88 [72, 108]	0.723
LV end-systolic volume, mL	126	41 [33, 55]	44 [32, 64]	41 [33, 55]	0.417
LVEF, %	128	53 [45, 56]	49 [36, 54]	53 [47, 56]	**0.015**
LVGLS, %	124	–12.3 [–15.0, –9.5]	–11.3 [–15.5, –9.1]	–12.4 [–14.9, –10.0]	0.421
Right ventricle					
RV wall motion Abnormality, n (%)	128	76 (59)	24 (100)	52 (50)	**<0.001**
RVGLS, %	125	–11.1 [–13.7, –8.2]	–8.9 [–12.0, –5.6]	–11.4 [–13.9, –8.4]	**0.007**
RVFWS, %	123	–10.9 [–14.6, –6.7]	–8.1 [–12.7, –5.6]	–11.6 [–14.9, –7.5]	**0.028**
TAPSE, mm	124	15.3 [12.0, 17.8]	17.5 [14.2, 20.3]	15.0 [11.3, 17.0]	**0.018**
Others					
IVC end-expiratory diameter, mm	124	17 [13, 20]	19 [17, 22]	16 [13, 20]	**0.007**
TRPG, mmHg	105	20 [16, 25]	19 [15, 22]	20 [16, 26]	0.120

Data are shown as the median [interquartile range] unless otherwise stated. IVC, inferior vena cava; LV, left ventricular; LVEF, left ventricular ejection fraction; LVGLS, left ventricular global longitudinal strain; RV, right ventricular; RVFWS, right ventricular free wall strain; RVGLS, right ventricular global longitudinal strain; TAPSE, tricuspid annular plane systolic excursion; TRPG, tricuspid regurgitation pressure gradient.

**Table 5:** Comparison of right ventricular function according to whether RVI was in the proximal or distal RCA

	N	Proximal RCA (n=21)	Distal RCA (n=3)	*p*-value
Right ventricle				
RVGLS, %	24	–9.0 [–12.2, –5.6]	–8.8 [–11.7, –3.3]	0.727
RVFWS, %	24	–8.3 [–12.5, –5.9]	–5.1 [–13.6, –4.0]	0.541
TAPSE, mm	24	17.6 [14.3, 20.3]	14.2 [7.6, 19.0]	0.185
Others				
IVC end-expiratory diameter, mm	22	19 [16, 21]	20 [17, 25]	0.472
TRPG, mmHg	19	19 [16, 21]	10 [8, 26]	0.433

Data are shown as the median [interquartile range]. IVC, inferior vena cava; RCA, right coronary artery; RV, right ventricular; RVFWS, right ventricular free wall strain; RVGLS, right ventricular global longitudinal strain; RVI, right ventricular infarction; TAPSE, tricuspid annular plane systolic excursion; TRPG, tricuspid regurgitation pressure gradient.
